# Wearable ultrasound and provocative hemodynamics: a view of the future

**DOI:** 10.1186/s13054-022-04206-7

**Published:** 2022-10-25

**Authors:** Jon-Émile S. Kenny, Chelsea E. Munding, Andrew M. Eibl, Joseph K. Eibl

**Affiliations:** 1 Health Sciences North Research Institute, 56 Walford Rd, Sudbury, ON P3E 2H2 Canada; 2 Flosonics Medical, Sudbury, ON Canada; 3 Northern Ontario School of Medicine, Sudbury, ON Canada

**Keywords:** Wearable ultrasound, Frank–Starling, Fluid responsiveness, Fluid tolerance, Venous Doppler

## Background

A new technology with exciting implications for critical care medicine was recently described by Wang and colleagues [1]. They report a bio-adhesive ultrasound (BAUS) with robust skin-coupling, comfort and excellent acoustic properties. In their feasibility study, they describe a small ultrasound transducer adhering over multiple tissues and organ systems, capable of acquiring 2-dimensional, brightness mode (B-mode) images in addition to pulsed wave and color flow Doppler. Furthermore, they reference a separate, fully contained, wireless, wearable, ultrasound system developed by our group [2]. We believe that both of these technologies may provide powerful, easy-to-use, hemodynamic measures for the twenty-first century intensivist [3]. In this *Comment* article, we briefly describe some significant physiological, clinical and practical implications of wearable ultrasound technology in the intensive care unit (ICU).

## Main text

### Physiological implications

Wang and colleagues characterize simultaneous common carotid artery and internal jugular vein B-mode images with the BAUS [1]. Specifically, they show that when a healthy subject shifts from upright-to-supine positions whilst wearing the BAUS, the jugular vein changes shape—from a collapsed to a distended profile. As Wang and colleagues note, this could act as a surrogate for changing right atrial pressure. Separately, the authors capture quantitative spectral Doppler ultrasound of the common carotid artery and its change from resting to post-exercise, demonstrating that the carotid artery velocity time integral (VTI) increases with exercise-induced cardiac output augmentation.

Though not explicitly described by Wang and colleagues, we believe what is most exciting about these data is *simultaneous, noninvasive* venous and arterial ultrasound surrogates for cardiac input and output, respectively. This novel paradigm has substantial inpatient and outpatient diagnostic and therapeutic implications—for example, real-time inferences of the Frank–Starling mechanism [2, 4–6]. This is of particular interest in the ICU. More specifically, during a provocative maneuver (e.g., passive leg raise (PLR) or rapid fluid challenge), increasing right atrial pressure coincident with little change in arterial flow intimates lost preload reserve [4], a feature typical of congestive heart failure [7] and septic shock [8]. While experts recommend confirming that right atrial pressure rises with a PLR to ensure that venous return challenges the Frank–Starling mechanism [9], this is cumbersome in practice. With wearable ultrasound technology, tracking these hemodynamic measures in real time, and in response to provocative maneuvers, is of tremendous utility to intensivists [10].

### Clinical implications

**Fig. 1:**
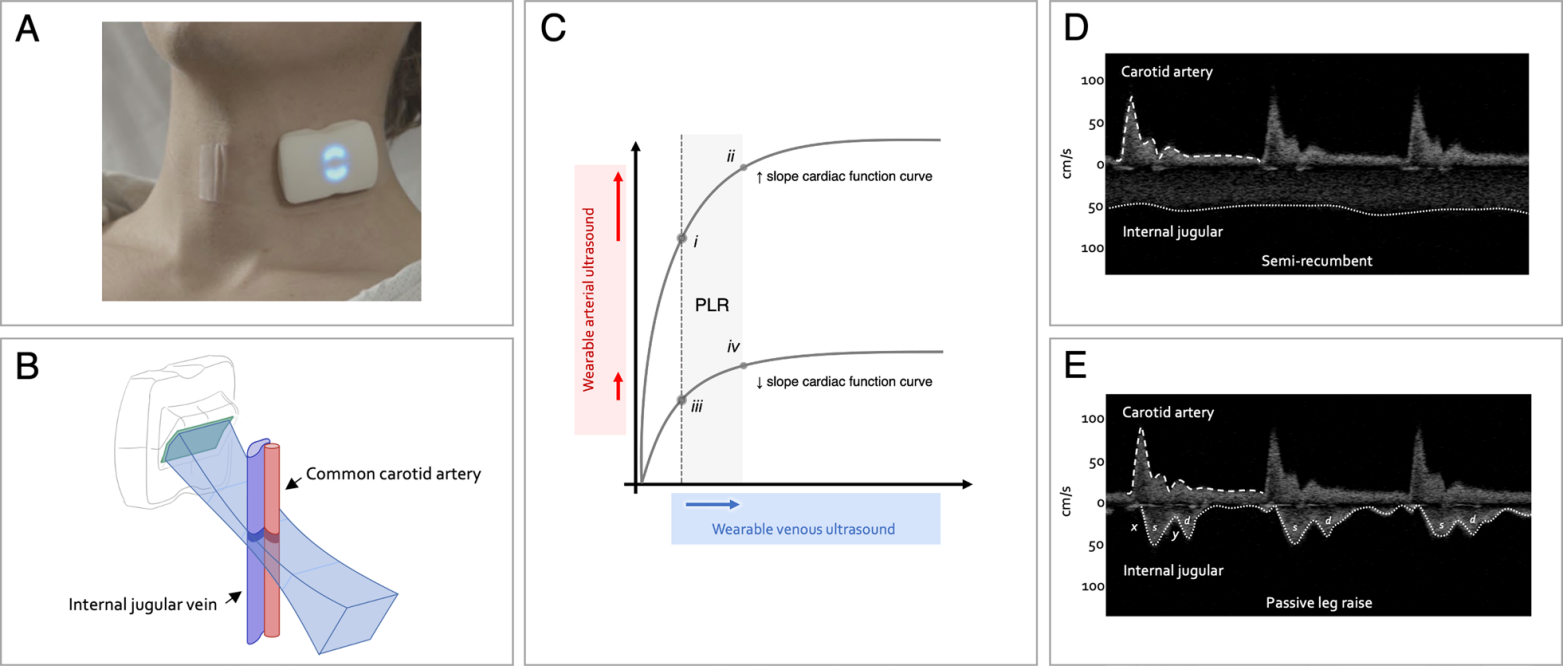
Wearable ultrasound and provocative hemodynamics. **A** Picture of a commercial wireless, wearable Doppler ultrasound system on a volunteer. **B** Graphical illustration of wearable ultrasound beam with simultaneous insonation of the common carotid artery and internal jugular vein. **C** Physiological framework showing how simultaneous venous and arterial ultrasound tracks the two axes of the Frank–Starling curve. Venous ultrasound tracks changing cardiac ‘input’ (e.g., right atrial pressure) and arterial ultrasound follows cardiac ‘output’ (e.g., stroke volume). Moving from point iii. to iv. illustrates a patient with low filling pressure who is fluid unresponsive [4]. **D** Simultaneous common carotid and internal jugular Doppler of a healthy volunteer in the semi-recumbent position. The jugular spectrum is non-pulsatile and higher velocity, suggesting a collapsed vein (i.e., low right atrial pressure). **E** Simultaneous common carotid and internal jugular Doppler of the same volunteer during PLR. The jugular velocity falls and becomes pulsatile, following the right atrial pressure waveform (i.e., the Doppler systolic ‘s’ wave is formed by the x-descent, and the diastolic ‘d’ wave is formed by the ‘y’ descent); this change is consistent with an enlarging jugular vein and right atrial pressure, and concomitantly, the carotid artery velocity time integral rises. This physiology is most compatible with moving from point i. to ii. in **C**

A recently published framework speculates how simultaneously acquired venous and arterial Doppler ultrasound could inform patient therapy and posits that Doppler ultrasound accompany all advanced critical care echocardiography to better delineate unique hemodynamic phenotypes [6]. For instance, *dynamic fluid intolerance* describes a hypo-perfused patient subtype with suggestive signs of low filling pressure (e.g., flat jugular vein, collapsing inferior vena cava, low central venous pressure) who is, nevertheless, found to be fluid unresponsive during a dynamic maneuver such as a PLR [6]. We envision how this phenotype could be detected using the ultrasound system described by Wang and colleagues. A BAUS worn over the apex of the left ventricle with a pulsed wave Doppler gate placed at the left ventricular outflow tract (LVOT) measures LVOT VTI in the baseline, semi-Fowler position; a separate BAUS on the neck simultaneously images a collapsed jugular vein. Then on PLR, the jugular vein is observed to distend (i.e., augmented right atrial pressure), while the LVOT VTI does *not* rise (i.e., stroke volume is unchanged). Thus, this patient who might have been mistakenly labeled as ‘fluid responsive’ or ‘fluid tolerant’ [11] based on signs of venous filling, is accurately determined to be ‘fluid unresponsive’ or ‘fluid intolerant’ following the provocative PLR maneuver. We have observed a similar phenotype using a wearable, continuous wave Doppler ultrasound system that records internal jugular and common carotid artery Doppler spectra as surrogates for changing right atrial pressure and stroke volume, respectively [4, 12] (Fig. 1).

### Practical implications

Wang and colleagues describe impressive coupling properties of their wearable BAUS. From a practical standpoint, we have observed that there needs to be a very good balance between firm skin adhesion and the ability to reposition. This is particularly important during signal acquisition because obtaining a good ultrasound window can be challenging and, in certain patient populations, requires multiple attempts to optimize signal. To account for these practical challenges, the fully contained, wireless, wearable Doppler ultrasound system described by our group is coupled to the skin using off-the-shelf ultrasound gel [2]. The ultrasound beam dimensions and properties were chosen specifically to favor simultaneous acquisition of Doppler spectra from the common carotid artery and internal jugular vein and not for anatomical flexibility, which can come at the cost of achieving an optimal signal. The device is affixed to the neck using a variety of adhesives, but when sealed, can maintain intermittent Doppler ultrasound scanning for multiple days and across a range of human activities.

## Conclusion

The recent work of Wang et al. offers a view of the future [1]. We expect that point-of-care ultrasound in the twenty-first century ICU will continue to miniaturize, become wearable and free itself from unwieldy tethers [13]. The new technologies described above could simultaneously and noninvasively acquire and transmit surrogates for cardiac input and output; we propose that this generates the slope of the cardiac function curve in real time. This paradigm could be an especially important supplement to functional hemodynamic monitoring [14] and an additional step toward personalized therapy in the ICU.

## Data Availability

Not applicable.

## Abbreviations

B-mode: Brightness mode
BAUS: Bio-adhesive ultrasound
ICU: Intensive care unit
VTI: Velocity time integral
PLR: Passive leg raise
LVOT: Left ventricular outflow tract
